# Infant Botulism: A Case Study in Integrated Clinical and Public Health Response

**DOI:** 10.1002/ccr3.71670

**Published:** 2025-12-09

**Authors:** John Gannon, Tiarnán Fallon Verbruggen, Sinéad Burke, Leah Loughlin, Cally Brits, Kara Tedford, John C. McHugh, Huw Mayberry, Mary O'Regan, Bridget Freyne, Niall Conroy

**Affiliations:** ^1^ Department of Public Health Health Service Executive Dublin Ireland; ^2^ Children's Health Ireland Crumlin Ireland; ^3^ Neurology Department Children's Health Ireland Crumlin Ireland; ^4^ Paediatric Intensive Care Children's Health Ireland Crumlin Ireland; ^5^ Pharmacy Department Children's Health Ireland Crumlin Ireland; ^6^ Department of Neurophysiology Children's Health Ireland Crumlin Ireland; ^7^ Paediatric Infectious Diseases Children's Health Ireland Crumlin Ireland; ^8^ Department of Paediatrics University of Oxford Oxford UK

**Keywords:** infectious diseases, pediatrics and adolescent medicine, public environmental & occupational health, public health

## Abstract

Early recognition of constipation, feeding difficulty, and descending weakness in infants is vital for prompt diagnosis of infant botulism. Bedside nerve studies support early treatment. Rapid public health notification and coordinated access to specific therapies, including timely use of BAT and BIG‐IV, are essential to optimize outcomes and safety.

## 1 Introduction

Infant botulism (IB) is an intestinal toxemia caused by the inhalation or ingestion of spores of *Clostridium botulinum* (or similar species), resulting in disruption of the neuromuscular junction and symmetric, descending flaccid paralysis [1]. *C. botulinum* is a gram‐positive, sporulating, obligate anaerobe found in soil. Spores can adhere to dust particles, as well as agricultural produce such as fruit, vegetables, and classically, honey [1]. *C. botulinum* cells produce one of eight serologically distinct neurotoxin types A–H, with emerging subtypes and dual‐toxin‐producing strains [2, 3, 4]. Other species, such as *Clostridium butyricum* and *Clostridium baratii* strains, have been shown to produce type E and F‐like neurotoxins, respectively [5, 6]. While each neurotoxin strain has a specific antitoxin, combination antitoxin preparations are commonly used [7].

IB differs from wound botulism or adult food‐borne botulism in its pathogenesis. Wound botulism occurs due to colonization of the wound with botulinum neurotoxin‐producing species of Clostridia that subsequently produce toxin, while food‐borne botulism involves ingestion of preformed toxin [3]. In IB, however, infants inhale or ingest spores that temporarily colonize the intestinal tract, germinate, and release botulinum neurotoxin (BoNT) into the bloodstream [1]. BoNT binds to peripheral cholinergic receptors at voluntary motor and autonomic neuromuscular junctions, with its light chains cleaving the proteins necessary for acetylcholine release from presynaptic terminals. This inhibits muscle contraction, resulting in hypotonia and flaccid paralysis [7]. Infants appear to be predisposed to this condition due to the immaturity of their intestinal microflora [1, 7]. While breastfed infants typically present later than formula‐fed infants, any protective role of breastmilk remains unclear [1].

IB is the most common cause of botulism in the United States, with 150–180+ cases annually [8, 9]. Cases have also been identified in Europe, South America, Asia, and Oceania, but at much lower incidence [5, 8, 10]. Reported cases range from 2 days to 72 weeks of age [1, 2, 7, 8]. The vast majority (91.2%) of cases in the United States occurred in infants under 6 months [1, 10, 11]. In Ireland, only three cases have previously been reported; one case due to toxin type A(B) and two caused by *Clostridium butyricum* type E neurotoxin following exposure to pet terrapin turtles [6, 10].

## 2 Case History/Examination

A previously healthy 9‐week‐old infant presented to a regional hospital with a 10‐day history of constipation and new difficulty breastfeeding. Overnight, the infant developed progressive oropharyngeal and facial weakness with loss of suck and gag reflex. Pupillary light reflexes became sluggish, and a symmetric descending flaccid paralysis evolved. The patient underwent a septic workup including blood culture and empiric intravenous antibiotic administration (cefotaxime and aciclovir) before being transferred to a pediatric intensive care unit for respiratory support and further investigation. This infant had been predominantly breastfed with some exposure to store‐bought anti‐colic drops and glycerine. The family lived on a farm, and a household member worked as a carpenter on local construction sites.

## 3 Methods

### 3.1 Investigations and Treatment

Serology, cerebrospinal fluid analysis, and extended viral panels revealed no infection or inflammation. MRI of the brain and spine was normal. Upon arrival at the tertiary pediatric facility, fecal samples obtained via rectal washout were sent to the Gastrointestinal Bacteria Reference Unit (GBRU) in London for botulinum culture and BoNT identification [12]. While awaiting results, bedside nerve conduction studies performed on day 3 and day 5 of hospitalization showed low‐amplitude compound muscle action potentials (CMAPs) with incremental increase on repetitive high‐frequency stimulation (50 Hz). These findings, together with the clinical picture, strongly supported a diagnosis of IB [1].

Following these findings, a consensus decision was made to treat the patient (on day 4 of hospitalization and onset of neurological symptoms) with Botulinum Antitoxin Heptavalent (A, B, C, D, E, F, G) [Equine] (BAT), the only readily available antidote in Ireland. Botulism Immune Globulin Intravenous [Human] (BIG‐IV) was also requested from the Infant Botulism Treatment and Prevention Programme (IBTPP) in California. *Clostridium botulinum* Type B was isolated from fecal samples by culture in *C. Botulinum* Isolation (CBI) media and enrichment broth. Nucleic acid extraction from the CBI broth is performed and screened for BoNT toxin gene types A, B, E & F using an in‐house Real‐Time PCR assay on Day 1, Day 3, and Day5/6. In addition, the enrichment broth is plated onto CBI plates to undergo anaerobic incubation. PCR assay for BoNT toxin genes A, B, E & F is performed on any lipase‐positive colonies for *C. botulinum* confirmation. The first positive result was reported and relayed to the clinical team on day 7 of the hospitalization, with BIG‐IV given later that day.

The patient experienced no adverse effects from BAT or BIG‐IV administration. Following the onset of fever and yellow endotracheal tube secretions on day 8, the patient was commenced on intravenous piperacillin‐tazobactam for 5 days. Gag reflexes were noted on day 10 with improvement in central tone. The patient was extubated on day 13 of hospitalization; 9 days following BAT administration and 6 days following BIG‐IV. They continued to receive multidisciplinary input from speech and language therapy, occupational therapy, physiotherapy, and dietetics. They were transferred to the ward on day 15 and discharged home with follow‐up on day 17. Follow‐up 27 days following presentation confirmed complete resolution of the patient's feeding ability and cry. The patient had developmental follow‐up with their local pediatric hospital.

### 3.2 Public Health Response

The pediatric infectious diseases team notified Public Health in September 2024, based on high clinical suspicion before laboratory confirmation. The case was categorized as probable IB, and a public health investigation was initiated without delay.

*C. botulinum* exists naturally in the environment. In most cases of IB, the source remains unidentified [1]. Potential sources include soil, dust, and environments where these may be disturbed (such as construction sites), as well as honey, peanut butter, and exposure to reptiles (such as terrapins) [13, 14, 15, 16, 17]. Because the incubation period is unclear, exposures in the 30 days prior to symptom onset are considered relevant [18, 19].

This infant was exclusively breastfed. Although they had not consumed honey, family members had, and other exposures included gripe water, a probiotic, a colic‐relief remedy, and a soother dipped in glycerine and aniseed. The infant had also received a course of oral omeprazole. It is worth noting the association between proton‐pump inhibitors and the effect they can have on the gut microbiome, with a reduction in diversity evident on stool culture [20, 21]. These microbiome changes have been linked with an increased risk of enteric infections in adult populations [20]. The family lived on a cattle farm, with recent visits to a zoo, but had no pets or direct animal exposures. The household water supply came from a private well, but the infant had not consumed this water. One family member worked in construction on a trout farm. There were no known sick contacts.

An incident management team was convened, including Public Health physicians, nurses, and epidemiologists, Pediatrics, Microbiology, Environmental Health, the Food Safety Authority of Ireland, and the Health Products Regulatory Authority. Samples of honey, gripe water, probiotic, and soother dip were submitted for *C. botulinum* testing at the GBRU in London [12]. Samples were tested for the presence of *C. botulinum* using the same method as described for the case's fecal samples, including environmental monitoring to exclude any potential environmental contamination from spores. Some items had been discarded by the family and were unavailable for testing. All available samples were tested and were negative for *C. botulinum* . The private well was contaminated with coliforms (but no *Clostridium* detected), and a boil water notice was issued pending remediation. No definite source was identified; contamination of work clothes from a family member working at a construction site was considered a possible route of exposure, although this cannot be proven.

## 4 Conclusion

This is the first case report that we are aware of in the literature describing both the clinical and public health response to a case of infant botulism (IB). From a clinical perspective, constipation in breastfed infants is often overlooked but may be a key early sign of IB [1, 3]. As the initial presentation can be mild and non‐specific, IB should be included in the differential diagnosis for lethargic infants. Bedside nerve conduction studies and electromyography are valuable adjuncts to aid the diagnosis. The case also highlights the importance of a multidisciplinary public health response to notifications of this nature. There is a need to consider a broad range of potential sources, including food, animal exposure, and environmental contaminants. This case underscores the value of rapid clinical recognition, early investigation and treatment, and coordinated public health action in the management of rare but high‐consequence conditions such as IB.

## 5 Discussion

This is the fourth reported case of infant botulism in Ireland and the first caused by type B botulinum neurotoxin. The case highlights the typical presentation and diagnostic features, particularly the importance of constipation as an early, often overlooked, symptom in breastfed infants [1]. Other presenting features include feeding difficulties, lethargy, and generalized weakness, progressing to bulbar palsy and symmetric descending paralysis [1, 7]. Infants may present with an expressionless face, bilateral ptosis, weak cry, poor head control, impaired gag, suck and swallow reflexes, as well as fatigable and sluggish pupillary reflexes [7]. Deep tendon reflexes may initially be normal, diminishing as paralysis advances. Respiratory and feeding support may be required [1].

The differential diagnosis includes infectious (e.g., sepsis, encephalitis, meningitis), metabolic or mitochondrial conditions, neuromuscular disorders (e.g., spinal muscular atrophy), hypothyroidism, and intoxication or poisoning [1, 7].

Laboratory confirmation is based on the identification of BoNT‐producing species of Clostridium in fecal samples using culture and neutralization bioassays [7]. Detection of toxin in serum is rare [1]. Most routine laboratory investigations are normal or reflect mild dehydration. Neuroimaging and lumbar puncture typically yield normal or nonspecific results [1, 7].

Bedside nerve conduction studies and electromyography can support the diagnosis and exclude alternative causes. Affected infants show reduced CMAP amplitude in at least two muscle groups, with an incremental increase on repetitive high‐frequency stimulation [7]. Absence of these findings, however, does not exclude the diagnosis [1, 7].

Early treatment with Botulism Immune Globulin Intravenous [Human] (BIG‐IV) is recommended by the American Academy of Pediatrics (AAP) and the IBTPP [3, 22]. BIG‐IV is derived from the pooled plasma from individuals immunized with the recombinant botulinum vaccine for serotypes A and B (rBV A/B) [22, 23]. It neutralizes free toxin and has a half‐life of 28 days. It has been shown to reduce mean hospital stay, intensive care duration, and duration of mechanical ventilation and feeding support [22, 24]. Early treatment (within 72 h of hospitalization) provides greater benefit [22, 24]. BIG‐IV is expensive (approximately $69,300 USD per dose) and must be sourced via the IBTPP in California, which can take several days depending on the patient's location [7, 22, 25].

Trivalent, pentavalent, or heptavalent equine‐derived botulism antitoxin (EqBA) treatment is preferred in adults, but due to risks such as anaphylaxis, serum sickness, and sensitivity to equine proteins, it is not routinely used in infants [3, 7]. Recently, a retrospective study in Argentina suggested EqBA may be a safe alternative where BIG‐IV is unavailable [26]. In that study, 31 cases of IB requiring supportive treatment with mechanical ventilation and who received EqBA were compared with 18 infants who did not receive the antitoxin. Mean length of stay in EqBA‐treated infants was shorter (28.7 ± 2.1 days versus 52.6 ± 6.8 days, *p*‐value 0.0007) [26]. Secondary outcomes including length of ICU stay, duration of mechanical ventilation, and duration of assisted feeding were also reduced [26]. Incidence of complications including secondary bacterial infection was lower in the treated group, and no adverse reactions or complications were reported as attributable to EqBA [26]. There are, however, no randomized controlled trials on the use or safety of EqBA in infants.

In Ireland, Botulinum Antitoxin Heptavalent (A, B, C, D, E, F, G) [Equine] (BAT) is the only antidote for IB that is readily available for immediate use. Previous Irish cases of *C. butyricum* type E infant botulism were also managed with BAT, followed by BIG‐IV for ongoing protection [6]. Early administration of BAT is associated with improved outcomes [1, 3, 6, 22]. In this case, the patient received both BAT and BIG‐IV without complication. BAT was given first due to its availability; however, the serotype half‐lives in equine‐derived antitoxin are very short (single vial: 7.5 –34.2 h) [27]. As patients with infant botulism can remain colonized for weeks to months, BIG‐IV was subsequently administered on the advice of the IBTPP. BIG‐IV, with its longer duration of action, is recommended to reduce the risk of relapse while intestinal colonization persists and prevent a potential rebound of symptoms [6, 8, 22, 28]. It should be noted that generally, safety data in infants are very limited.

No specific source was identified for this case, consistent with most IB reports. While honey is the best‐known source, exposures can include dust or soil, reptiles, peanut butter, and soiled clothing from construction environments [15, 18]. One case has been reported in Japan due to an untreated well‐water source that likely became contaminated with spores from soil [29]. The rarity of IB in Ireland led to the development of an *aide memoire* for public health physicians in collaboration with the National Health Protection Directorate to facilitate rapid action in the case of future incidences. Additionally, the National Healthy Childhood Programme has now provided online advice to parents around the risk of soiled construction apparel and infant infection [30]. While it is impossible to eliminate dust and soil, and therefore spores, from the infant's surrounding environment, this guidance aims to promote awareness and recognition of a rare but potentially fatal toxemia.

## Author Contributions

**John Gannon:** conceptualization, data curation, formal analysis, investigation, writing – original draft, writing – review and editing. **Tiarnán Fallon Verbruggen:** conceptualization, data curation, formal analysis, methodology, writing – original draft, writing – review and editing. **Sinéad Burke:** investigation, writing – review and editing. **Leah Loughlin:** investigation, writing – review and editing. **Kara Tedford:** methodology, writing – review and editing. **John C. McHugh:** investigation, writing – review and editing. **Huw Mayberry:** investigation, methodology, writing – review and editing. **Mary O'Regan:** investigation, writing – review and editing. **Cally Brits:** investigation, writing – review and editing. **Bridget Freyne:** data curation, formal analysis, investigation, methodology, project administration, supervision, writing – original draft, writing – review and editing. **Niall Conroy:** conceptualization, data curation, formal analysis, investigation, methodology, project administration, supervision, writing – original draft, writing – review and editing.

## Funding

The authors have nothing to report.

## Ethics Statement

The authors have nothing to report.

## Consent

Written informed consent from the patient's legal guardian was obtained according to journal guidelines. All identifying information has been anonymised to protect patient confidentiality.

## Conflicts of Interest

The authors declare no conflicts of interest.

## Data Availability

The data that support the findings of this study are available on request from the corresponding author. The data are not publicly available due to privacy or ethical restrictions.
